# Magnitude and associated factors of urinary tract infections among adults living with HIV in Ethiopia. Systematic review and meta-analysis

**DOI:** 10.1371/journal.pone.0264732

**Published:** 2022-04-01

**Authors:** Molla Yigzaw Birhanu, Samuel Derbie Habtegiorgis, Wodaje Gietaneh, Simegn Alemu, Tesfa Birlew Tsegaye, Getamesay Molla Bekele, Abtie Abebaw, Tebelay Dilnessa, Haymanot Tewabe Elmneh, Haile Amha, Daniel Bekele Ketema, Tsige Gebre Anto, Melaku Desta, Selamawit Shita Jemberie

**Affiliations:** 1 Department of Public Health, College of Health Science, Debre Markos University, Debre Markos, Ethiopia; 2 Department of Gynecology and Obstetric, School of Medicine, Debre Markos University, Debre Markos, Ethiopia; 3 Department of Medical Laboratory Science, College of Health Science, Debre Markos University, Debre Markos, Ethiopia; 4 Department of Nursing, College of Health Science, Debre Markos University, Debre Markos, Ethiopia; 5 Department of Midwifery, College of Health Science, Debre Markos University, Debre Markos, Ethiopia; King Abdulaziz University, SAUDI ARABIA

## Abstract

**Background:**

Urinary tract infection is a major public health problem in developing countries among immunocompromized populations where there are limited health-care services. People living with human immunodeficiency virus (HIV) are more likely to develop urinary tract infections (UTI) due to the suppression of their immunity. There is no single representative figure as well as the presence of significant heterogeneity among studies conducted on people living with HIV in Ethiopia. Hence, this study tried to pool the magnitude of UTI among people living with HIV in Ethiopia.

**Method:**

To find relevant studies, researchers looked through Web of Science, Science Direct, PubMed, EMBASE, the Cochrane Library, Google Scholar, and Worldwide Science. The I^2^ statistic was used to examine for heterogeneity among the studies that were included. To evaluate the pooled effect size across studies, a random-effects model was used. The presence of publication bias was determined using a funnel plot and Egger’s regression test. STATA^TM^ version 14.0 software was used for all statistical analyses.

**Results:**

A total of 7 studies with 2257 participants were included in this meta-analysis. UTI was shown to be prevalent in 12.8% (95% CI: 10.8–14.79, I^2^ = 50.7%) of HIV patients. Being male (0.35, 95% CI:0.14, 1.02), rural residents(OR:1.41,95% CI: 0.85, 2.34), no history of catheterization (OR: 0.35, 95% CI: 0.06, 1.85), had no history of DM (OR:0.84, 95% CI:0.12, 0.597) and having CD4 count greater than 200 (OR:0.36 95% CI: 0.06, 2.35) were the factors which were the associated factors assessed and having association with UTI among people living with HIV but not statistically significant.

**Conclusions:**

In Ethiopia, one in every eight HIV-positive people is at risk of acquiring UTI. Regardless, we looked for a link between sex, residency, CD4, catheterization history, and DM and UTI, but there was none. To avoid this phenomina, every HIV patient should have a UTI examination in every follow-up.

## Introduction

Urinary tract infection (UTI) is caused by the bacterial invasion which can multiply in the urinary tract system [1]. The burden of UTI is increasing among people living with HIV due to the immunocompromization secondary to the viral infection and the immune system can no longer fight against invading bacteria and resulting bacteriuria [2]. It is commonly classified as symptomatic and asymptomatic and more than three-fifth (70%) of cases are caused by asymptomatic bacteriuria (ASB) which needs treatment but left untreated it causes about 40% cystitis and 30% pyelonephritis [3, 4]. Many gram positive and negative bacteria cause urinary tract infection among adults living with HIV [5, 6]. Those gram-negative bacteria cause UTI are like Escherichia coli (60–70%), Proteus mirabilis (5–10%), Klebsiella pneumoniae (10%) and Pseudomonas aeruginosa (2–5%) and gram positive bacteria like Staphylococci (saprophyticus and aureus), Streptococcus pneumonia, Haemophilus influenza, Citrobacter freundii, and Enterococcus faecalis [1, 7–9].

The predisposing factors to UTI are low socioeconomic status, increasing age, urinary tract anomalies, previous treatment for UTI, other medical conditions like diabetes, and immune compromised condition like HIV/AIDS and spinal cord injuries [10].

Improving knowledge on the prevalence, spectrum of bacterial uropathogen, and associated factors of urinary tract infections among pregnant women living with HIV could substantially improve the current diagnostic and treatment guidelines for better prognosis of pregnant women living With HIV. For policy maker and program planner, it is important to have insight or information on the magnitude, spectrum of bacterial uropathogen, and factors associated with urinary tract infection among pregnant women living with HIV.

Although various primary studies have showed that the frequency of UTI among HIV patients is high and treatment choices are limited, their findings have revealed significant variance in the prevalence of UTI in Ethiopia. Given this, it is critical to thoroughly comprehend the extent of the UTI problem in the region. As a result, the goal of this research was to determine the overall prevalence of UTI and its associated factors among HIV patients in Ethiopia. This discovery provides a scientific foundation for a better understanding of the prevalence of UTI in HIV patients and aids in the development of effective prevention methods.

## Methods

### Data source and search strategy

We searched the Web of Science, Science Direct, PubMed, EMBASE,Cochrane Library, Google Scholar, Addis Ababa digital library, and Worldwide Science databases for literature on the topic. Up until August 18, 2021, the condition, context, and population keywords were merged using Boolean terms to search the Pubmed database for published reports of urinary tract infection among people living with HIV in Ethiopia. As a result, after the two authors (MYB and MD) searched the source articles independently using the following search keywords and MeSH phrases paired with Boolean words, (((("adult"[MeSH Terms] OR "adult"[All Fields] OR "adults"[All Fields] OR "adult s"[All Fields]) AND "people living with hiv"[Text Word]) OR (("anti retroviral agents"[Pharmacological Action] OR "anti retroviral agents"[MeSH Terms] OR ("anti retroviral"[All Fields] AND "agents"[All Fields]) OR "anti retroviral agents"[All Fields] OR "antiretroviral"[All Fields] OR "antiretrovirally"[All Fields] OR "antiretrovirals"[All Fields]) AND "user"[Text Word]) OR ((("people s"[All Fields] OR "peopled"[All Fields] OR "peopling"[All Fields] OR "persons"[MeSH Terms] OR "persons"[All Fields] OR "people"[All Fields] OR "peoples"[All Fields]) AND ("lived"[All Fields] OR "lives"[All Fields] OR "living"[All Fields] OR "livings"[All Fields])) AND "HIV"[MeSH Terms])) AND (("urinary tract infection"[Text Word] OR "urinary tract infections"[Text Word] OR "UTI"[Text Word] OR "Bacteriuria"[Text Word] OR "urinary tract infections"[MeSH Terms]) AND "Ethiopia"[Text Word])) OR "Ethiopia"[MeSH Terms] (Table 1), the presence of conflicts between two authors searching articles were resolved by discussing and reaching on an agreement in the presence of a third author (GMB). MYB and SDH used conventional Microsoft Excel spreadsheets adapted from JBI to extract data from the included papers, which were then imported into STATA^TM^ version 14.0 for further management and analysis. The Preferred Reporting Items for Systematic Reviews and Meta-Analyses (PRISMA) were used to format this systematic review and meta-analysis [11].

**Table 1:** Pubmed searching history.

Search	Search terms	Items
**1**	**(((((((Urinary tract infection[Text Word]) OR (Urinary tract infections[Text Word])) OR (UTI[Text Word])) OR (Bacteriuria[Text Word])) OR (Urinary tract infection[MeSH Terms]) Condition**	**16,437**
**2**	**((((((((Adult people living with HIV[Text Word])) OR (Antiretroviral user[Text Word])) OR (people living with HIV[MeSH Terms])) Population**	**275**
**3**	**(((("adult"[MeSH Terms] OR "adult"[All Fields] OR "adults"[All Fields] OR "adult s"[All Fields]) AND "people living with hiv"[Text Word]) OR (("anti retroviral agents"[Pharmacological Action] OR "anti retroviral agents"[MeSH Terms] OR ("anti retroviral"[All Fields] AND "agents"[All Fields]) OR "anti retroviral agents"[All Fields] OR "antiretroviral"[All Fields] OR "antiretrovirally"[All Fields] OR "antiretrovirals"[All Fields]) AND "user"[Text Word]) OR ((("people s"[All Fields] OR "peopled"[All Fields] OR "peopling"[All Fields] OR "persons"[MeSH Terms] OR "persons"[All Fields] OR "people"[All Fields] OR "peoples"[All Fields]) AND ("lived"[All Fields] OR "lives"[All Fields] OR "living"[All Fields] OR "livings"[All Fields])) AND "HIV"[MeSH Terms])) AND (("urinary tract infection"[Text Word] OR "urinary tract infections"[Text Word] OR "UTI"[Text Word] OR "Bacteriuria"[Text Word] OR "urinary tract infections"[MeSH Terms]) AND "Ethiopia"[Text Word])) OR "Ethiopia"[MeSH Terms]**	**14,960**

### Eligibility criteria

We used the following criteria to include studies for this study: (1) observational studies that reported a prevalence of UTI among HIV/AIDS patients in Ethiopian adults; (2) articles published in peer-reviewed journals or grey literature; and (3) articles published in English between the start of the study and August 18, 2021. We excluded studies if (1) they were not fully accessible and (2) case series conducted on urinary tract infection among adult Ethiopian living with HIV.

### Operational definition

#### UTI

Is the presence of pathogenic bacteria in considerable numbers within the urinary system (≥ 10^5^ CFU/ML) [12].

### Outcome of interest

There are two key outcomes of interest in this study. The first was the prevalence of urinary tract infection among HIV-positive adults, and the second was the associated factors of urinary tract infection among HIV-positive adults in Ethiopia, both of which were described in the original study. Asymptomatic bacteriuria is described as the presence of a substantial amount of bacteria in the urine despite the absence of clinical signs or symptoms of a urinary tract infection [13].

### Study selection

During searching in the advanced pubmed, all the identified articles were uploaded into EndNote version 8.2, and duplications were removed. Screening by titles and abstracts were done by two reviewers (MYB and MD) for assessment against the inclusion criteria for the review. The full text of the selected article was assessed in detail against the inclusion criteria by two reviewers (MYB and MD). After reading of the full text, studies were excluded in this systematic review and Meta analysis since which did not meet the inclusion criteria were recorded and reported in the systematic review. The presence of disagreements between the reviewers were resolved through discussion in the presence of third researcher (GMB).

### Data extraction and quality assessment

After identifying the papers to be included, two authors (MYB and SDH) extracted the data using a Microsoft spreadsheet. The first/corresponding author, publication year, region, study design, sample size, data collecting period, sampling procedure, prevalence of UTI with its 95 percent confidence interval (CI), and associated factors (sex, residence, CD4 count, history of catheterization and diabetes millitus) were all extracted from each included study. In the presence of the third author (GMB), the consistency of the extracted data by the two authors was ensured, and any disagreements during data extraction were resolved through group consensus. The methodological quality of each included study was assessed using the Newcastle–Ottawa Scale [14]. Representativeness, response rate, method of measuring outcomes, subject comparability, and the suitability of the statistical test used to evaluate the data are all assessed using this instrument. Any conflicts concerning each article were resolved collaboratively by all authors, who each presented their point of view, and the final decision was achieved by consensus (S1 File).

### Risk of bias assessment

All included studies were subjected to a risk of bias evaluation established by Hoy et al. [15] to examine the external and internal validity of nonrandomized studies in meta-analyses. The Hoy score is a ten-point scale that categorizes research as "high risk of bias" (total score ≤4), "moderate risk of bias" (total score 5–7), or "low risk of bias" (total score 8–10) (S2 File). The risk of bias assessment of the included studies was done by two authors (MYB& GMB).

### Heterogeneity and publication bias

To investigate heterogeneity between studies, Cochran’s Q and I^2^ statistics were used [16], which estimates the percentage of total variation across studies due to true differences between-studies rather than chance, with I^2^ values of 25, 50, and 75 percent, respectively, representing low, medium, and high heterogeneity. Subgroup analysis and Meta-regression analysis were used to look into the origins of heterogeneity. Each study’s effect on the overall prevalence was also subjected to a sensitivity analysis. Visual inspection of funnel plots and the objective use of Egger’s test were used to determine publication bias [17].

### Data management and analysis

Because we expected significant differences in UTI prevalence estimates among the included studies, we utilized the DerSimonian–Laird random-effects models to generate the pooled prevalence of UTI [18]. The presence and absence of heterogeneity across studies was assessed using I^2^ and Q tests. A forest plot was used to calculate the pooled effect size (i.e., prevalence) with a 95 percent confidence interval (CI). STATA^TM^ version 14.0 software was used for all statistical analyses [19]. The PRISMA checklist is used to report all of the findings (S3 File). To identify the source of heterogeneity, subgroup analysis and meta regression were performed. To identify the factors associated with UTI, sex, residence, CD4 count, history of catheterization and DM were performed using meta-regression analysis.

## Results

### Selection of the studies

**Fig 1:**
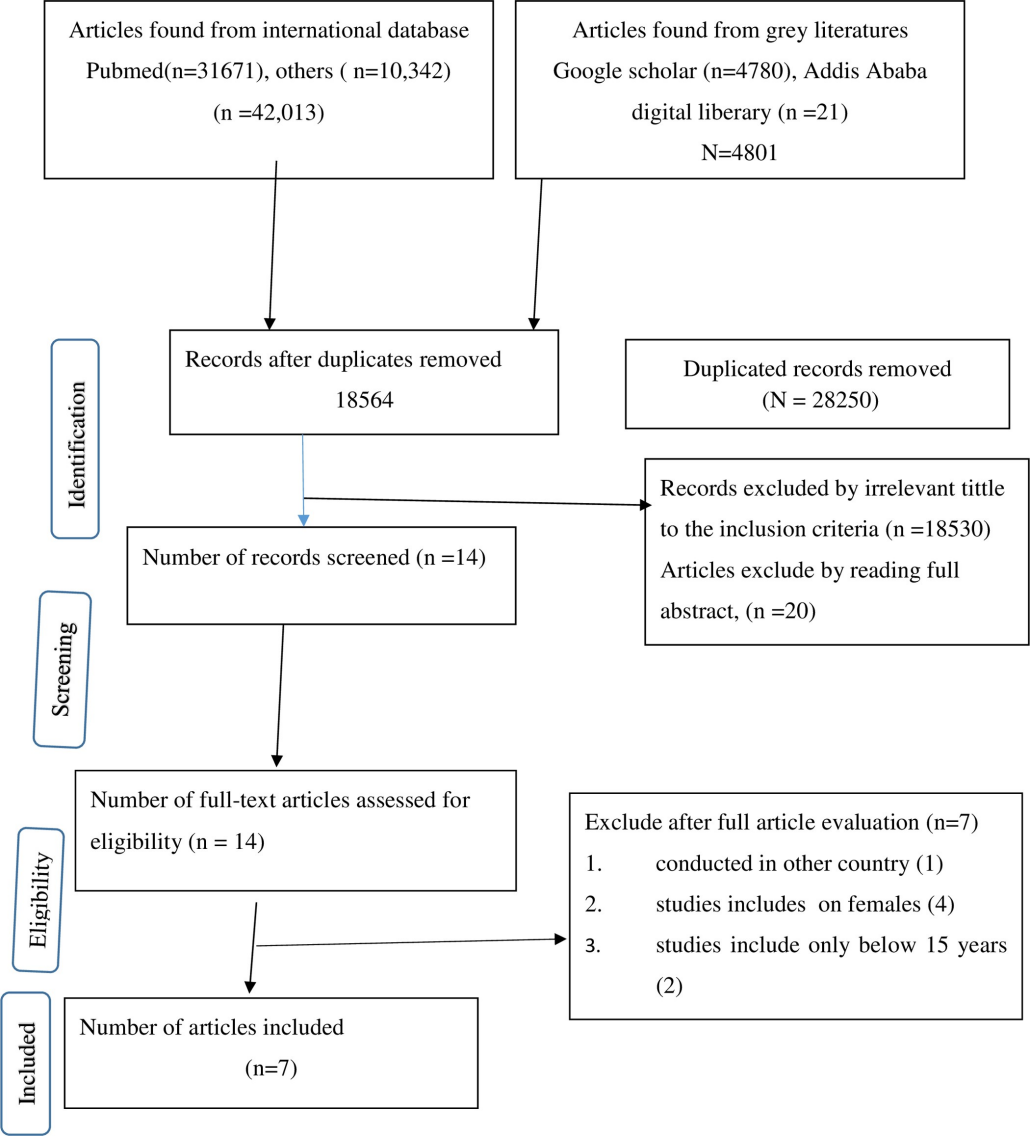
PRISMA flow diagram for selection of the included studies.

The initial search identified 46814 articles, which were catalogued in citation management software (EndNote X 8.2). Of these, 31671 studies were retrieved from PubMed, Web of Science (3500), Google Scholar (4780), Addis Ababa digital library (21), Science Direct (1250), and EMBASE (2000), Cochrane Library (1590), and Worldwide Science databases (2002). Of them, 28250 duplicate records were identified and removed. Following removal of duplicate studies, the titles and abstracts were evaluated, and 18550 studies were excluded based on the prespecified inclusion criteria. Then, 14 studies were included for further assessment. After reviewing the full text, four studies conducted out of Ethiopia and among pregnant women [20–23], one study conducted out of Ethiopia [5, 24] and one study conducted among children and out of Ethiopia [25] were excluded and 7 articles [26–31] were included for the final analysis (Fig 1).

### Baseline characteristics of the study participants

The study contains seven studies that enrolled a total of 2257 people and were conducted between 2013 and 2020. They were all used to calculate the combined prevalence of UTI among HIV-positive persons in Ethiopia [26–31]. All of the studies that were considered were cross-sectional in design. Males and females ≥ 18 years old took part in the investigations. The number of people that took part in each study ranged from 165 to 467. The frequency of UTI in HIV patients was determined in several Ethiopian regions; one study from Amhara [31], Two from Addis Ababa [28, 30], two from Oromia [27, 29], and two from SNPR [26, 32]. With regard to the sampling technique, three studies [26, 31, 32] used simple random sampling, one study used systematic random sampling, three studies [29], two studies [28, 30] used convenience sampling, and one study [27] used the conscutative sampling method.

### Urinary tract infection

**Fig 2:**
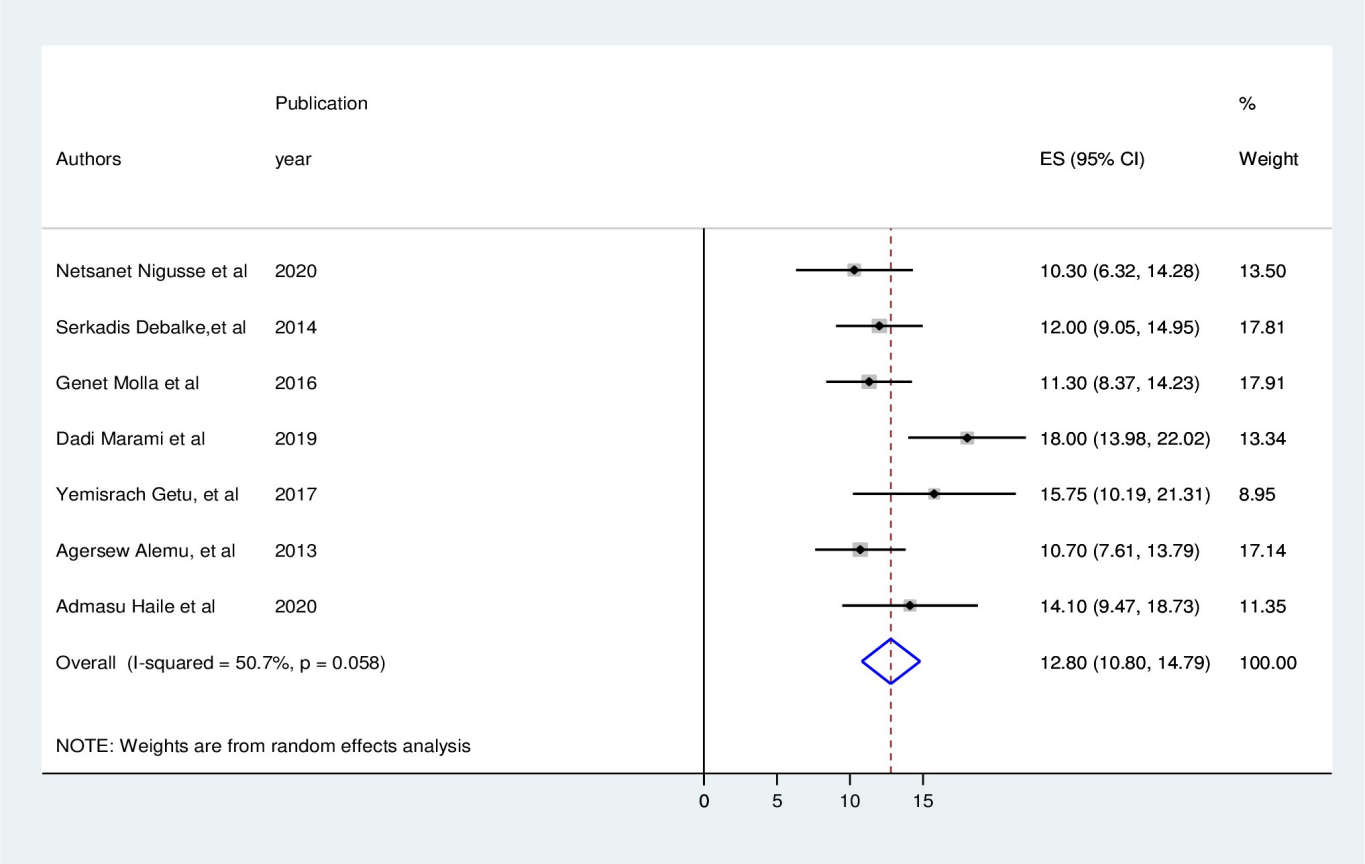
The pooled prevalence of urinary tract infection among people living with HIV in Ethiopia.

According to the current meta-analysis, the overall prevalence of UTI among people living with HIV in Ethiopia was 12.8% (95% CI: 10.8–14.79, I2 = 50.7%) (Fig 2).

### Subgroup analysis

**Fig 3:**
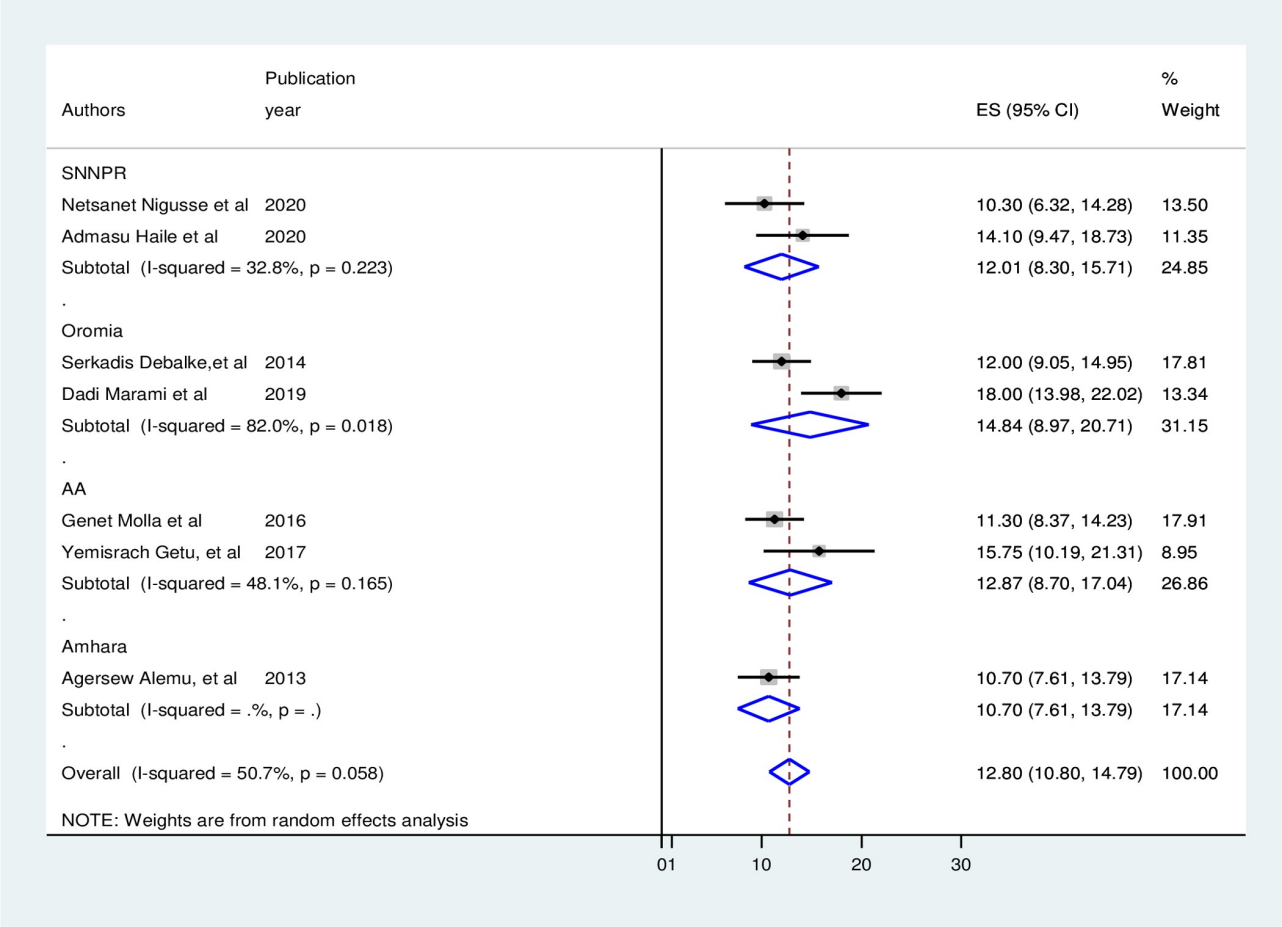
Subgroup analysis over region.

**Fig 4:**
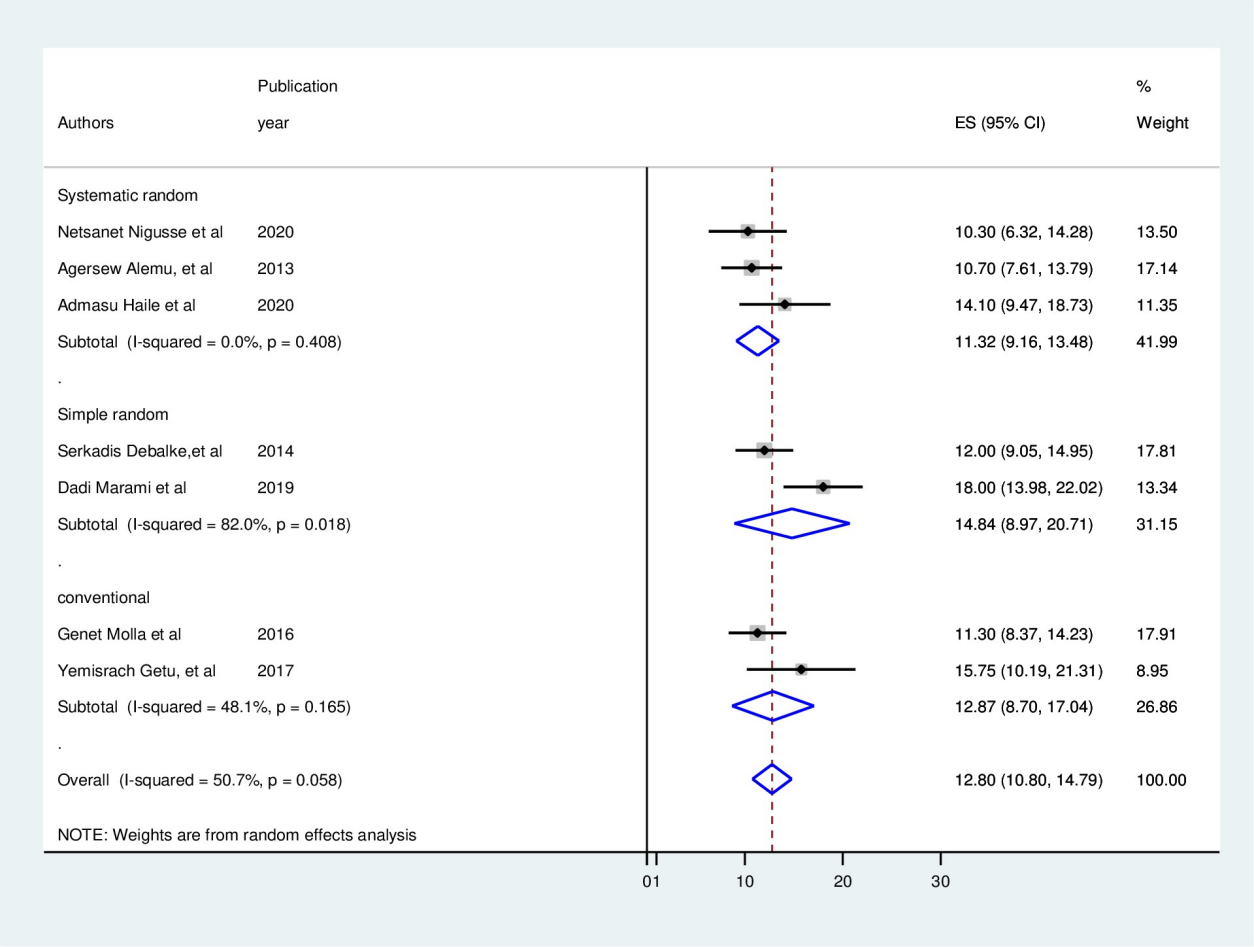
Subgroup analysis over sampling method.

Subgroup analysis deployed publication year, regions, sample size, sampling method, and study period was used to find the cause of heterogeneity across the included studies. According to the results of the subgroup analysis, the pooled prevalence of UTI among HIV-positive people ranged from 10.7% in Amhara to 14.84% in Oromia. So the Oromia region had the highest pooled prevalence of UTI, followed by Addis Ababa with 12.78, and Amhara with the lowest pooled prevalence (Fig 3). Simple random sampling had the largest heterogeneity (OR:14, 95% CI: 8.97–20.7, I^2^ = 82%, p = 0.002), followed by conventional sampling (OR: 12.87, 95% CI: 8.7–17.04, I^2^ = 48%, p = 0.165) in terms of sample method (Fig 4).

### Meta-regression analysis

Meta-regression analysis was used to determine the cause of heterogeneity for UTI among people living with HIV by taking into account publication year,sampling method, study period, region and sample size. However, our findings revealed that none of the covariates were statistically significant in predicting the presence of heterogeneity (Table 2).

**Table 2:** Checking publication bias using egger test.

Std_Eff	Coef.	Std. Err.	t	P>|t|	[95% Conf. Interval]
slope	5.86	4.06	1.44	0.209	-4.58, 16.31
Bias	3.69	2.21	1.67	0.156	-1.99, 9.38

### Publication bias

**Fig 5:**
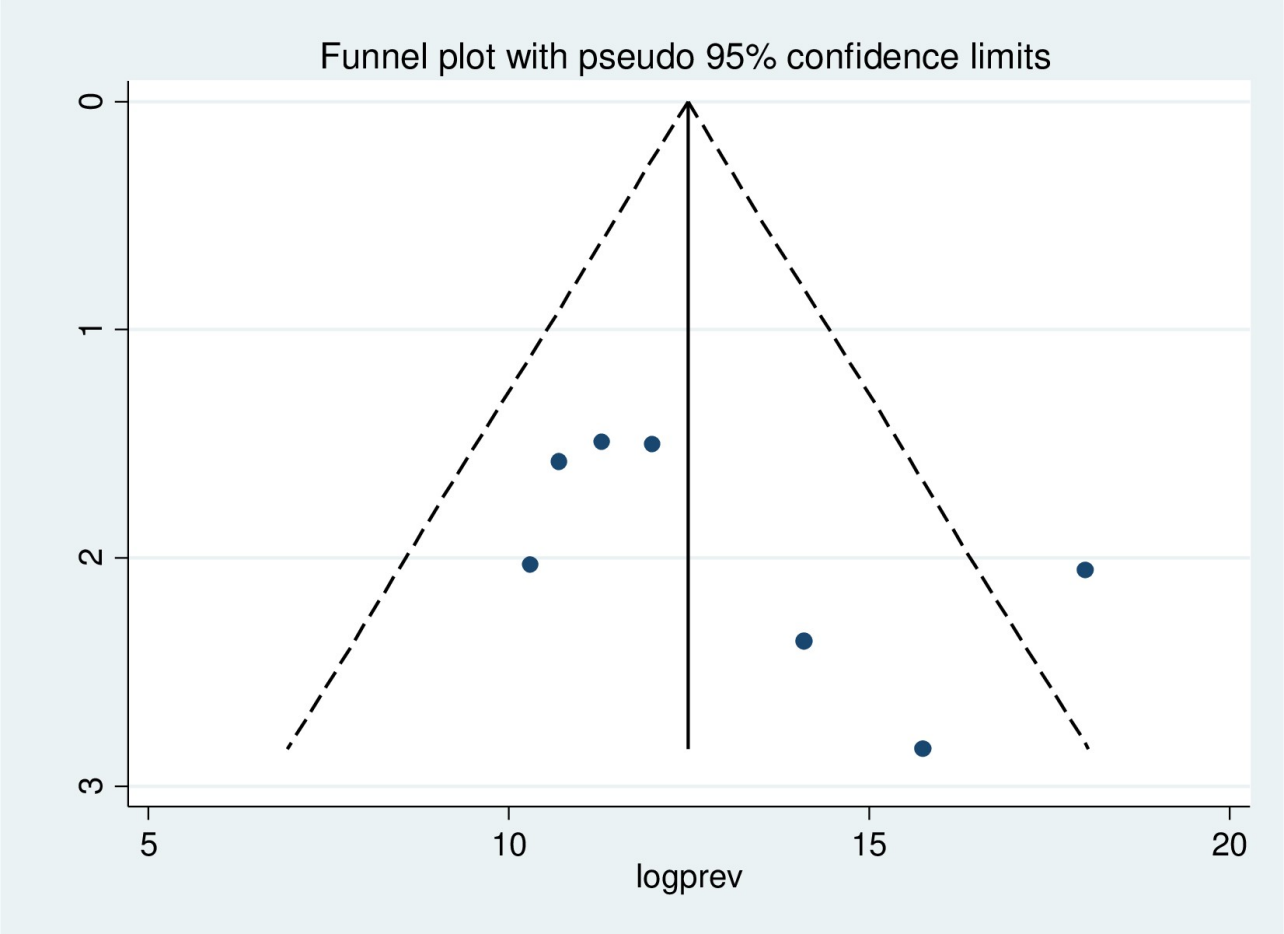
Funnel plot to test publication bias of the 7 studies.

When UTI among HIV-positive patients was analyzed, as shown in Fig 5, a visual evaluation of the funnel plot revealed that there was no publication bias among the included seven studies, as seen by the symmetrical distribution of the funnel plot. Similarly, the outcome of Egger’s test for the presence of publication bias was not statistically significant (P = 0.156) too (Table 1).

### Factors associated with urinary tract infection

**Fig 6:**
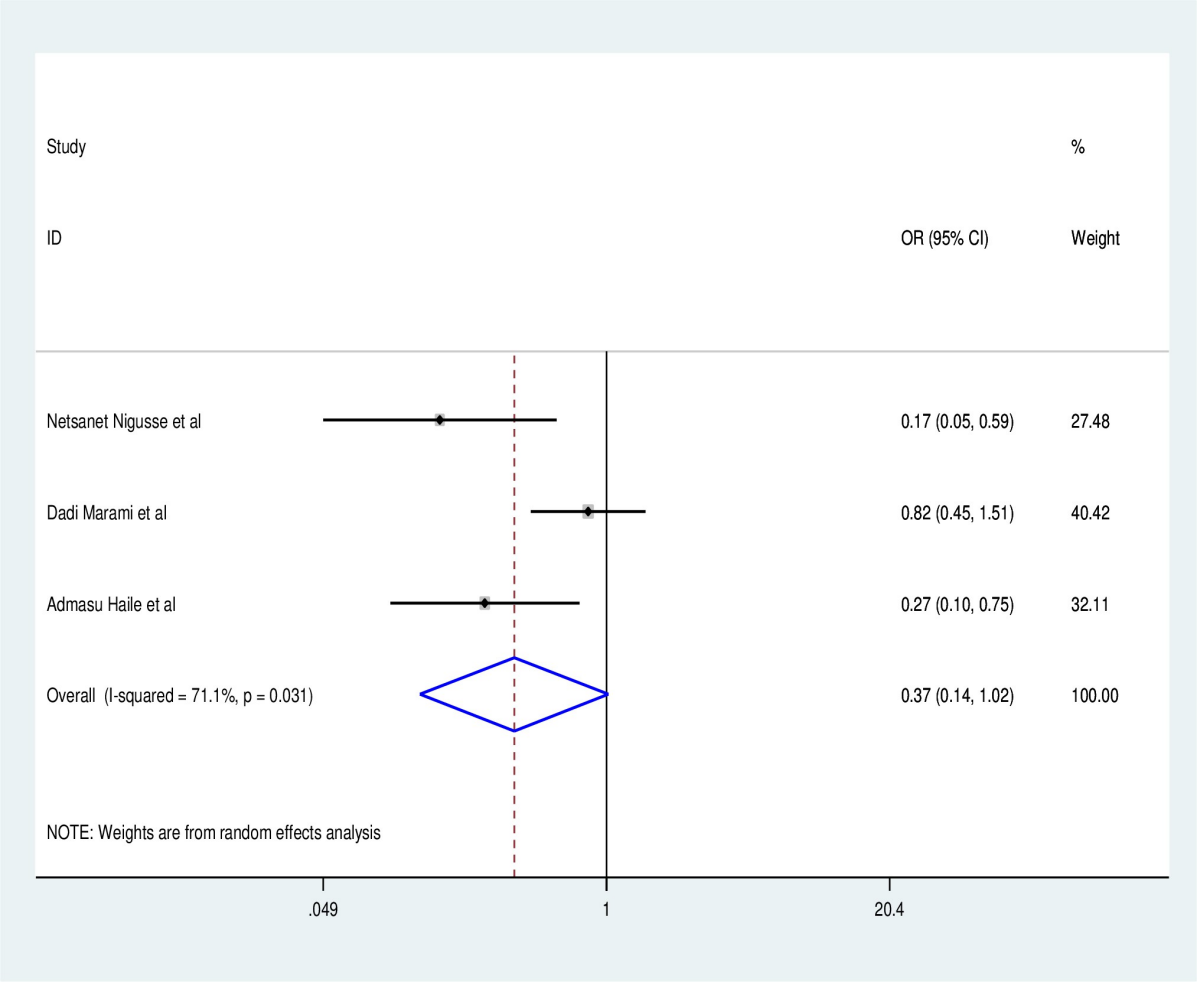
Factor analysis over sex.

**Fig 7:**
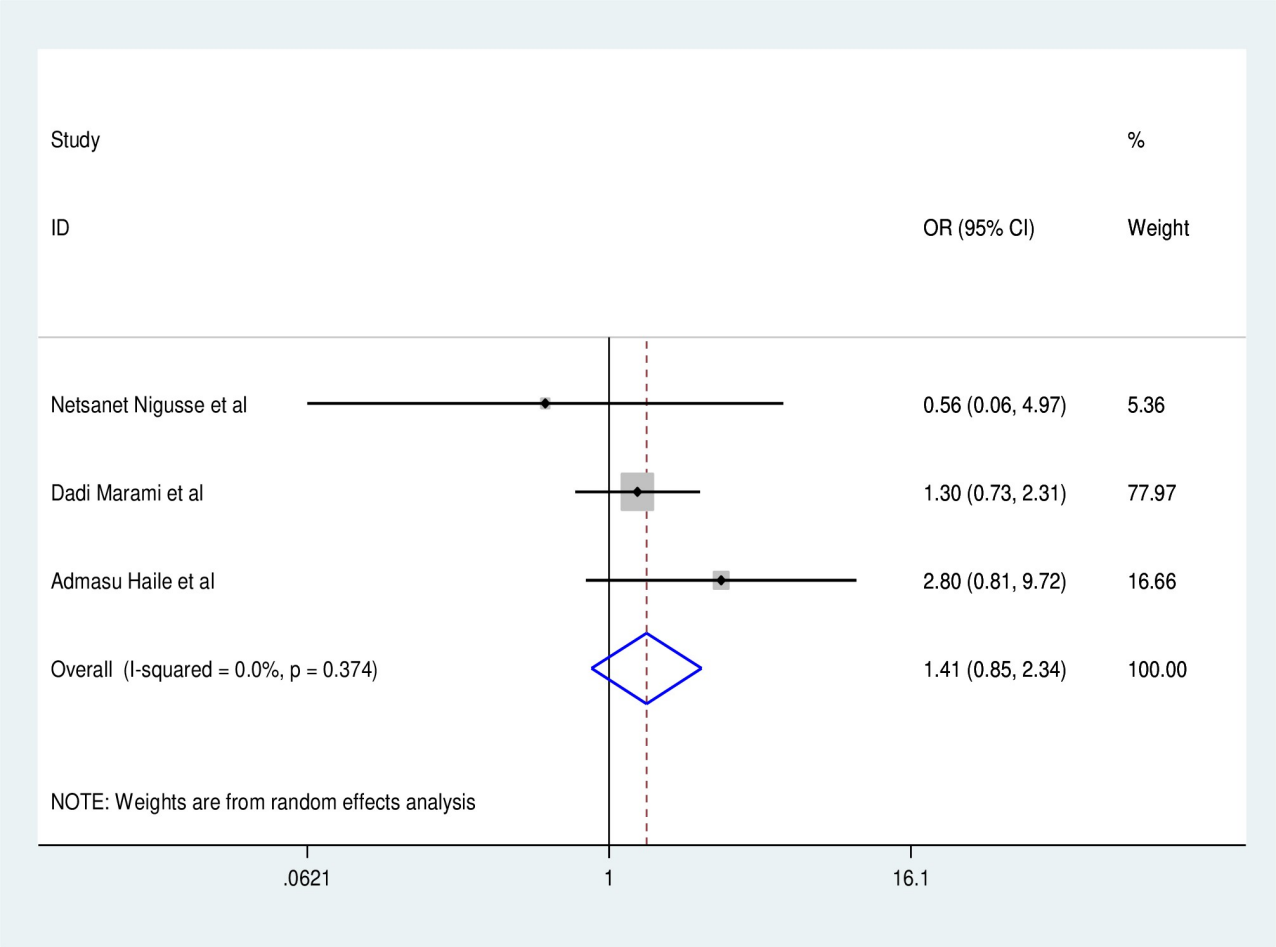
Factor analysis over resident.

Based on this systematic review and Meta-analysis, UTI among people living with HIV in the Ethiopian context was done on sex, CD4 count, residence, history of catherization, and history of diabetes milletus. To determine the factors associated with UTI among people living with HIV in Ethiopia three studies [26, 29, 32] were included. As a result, the pooled effect of three studies revealed that males living with HIV have a lower risk of UTI than females (OR:0.35, 95% CI: 0.14, 1.02), with statistically significant heterogeneity across studies (I^2^:71.1%, P = 0.03) (Fig 6). Rural residents are more likely to be exposed to UTI than urban residents (OR:1.41, 95% CI: 0.85, 2.34), with no statistically significant difference across studies (I^2^: 0.00, P = 0.37) (Fig 7).

**Fig 8:**
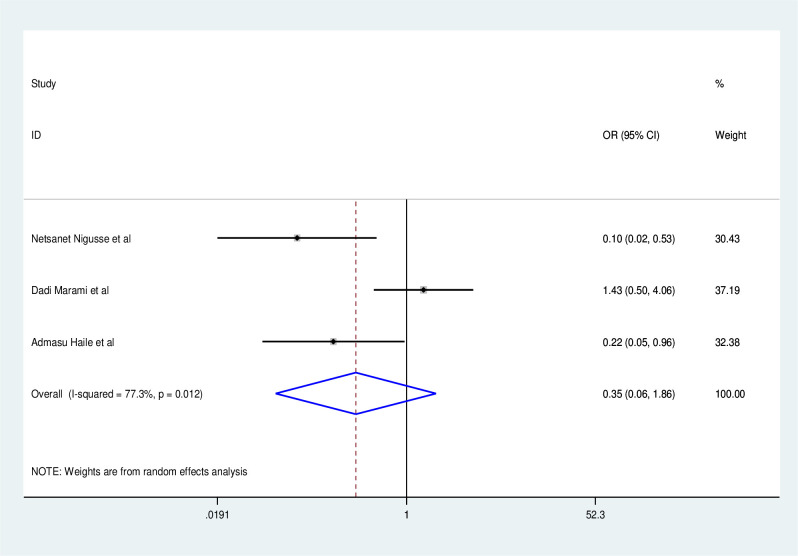
Factor analysis.

**Fig 9:**
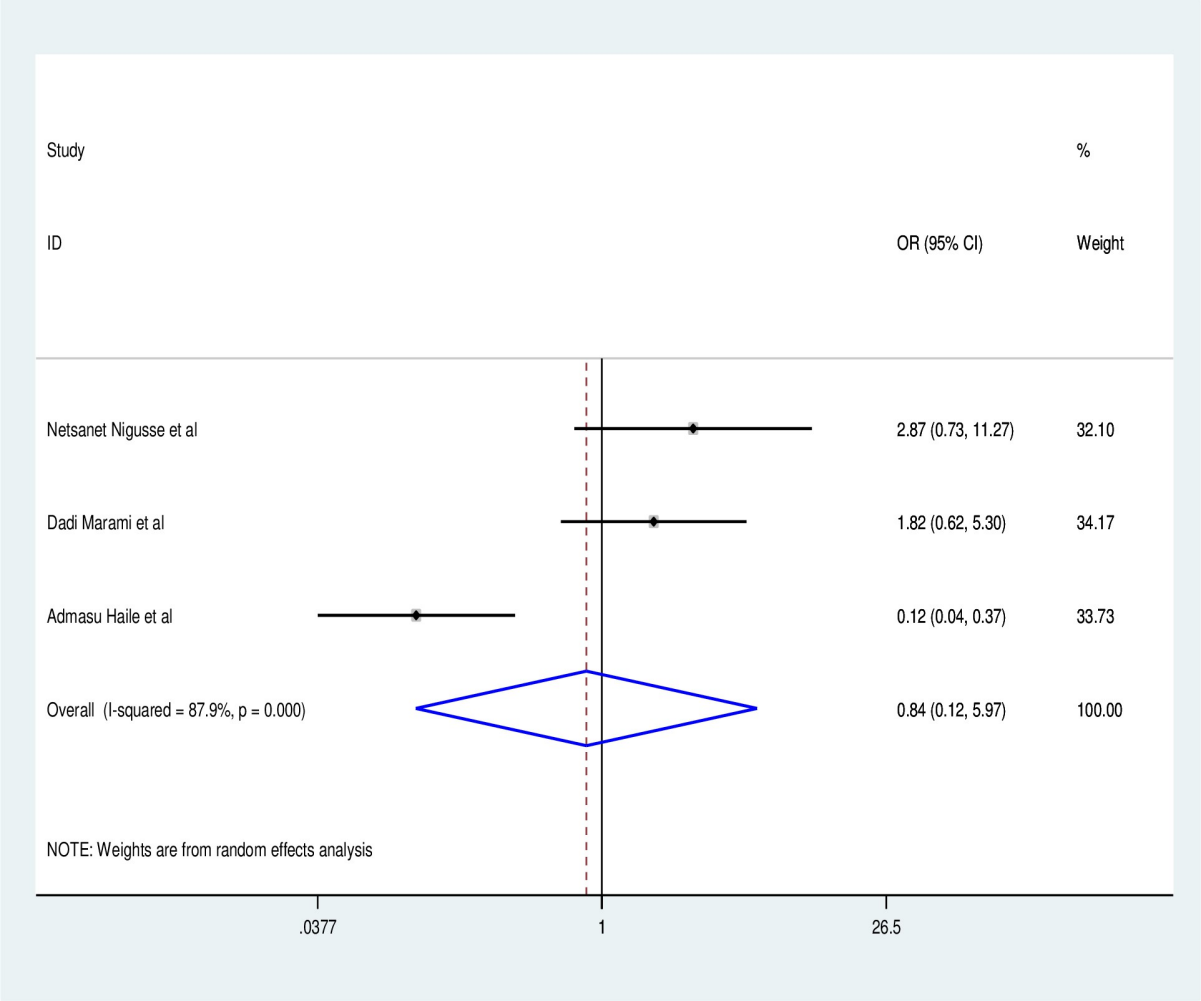
Factor analysis over catheterization.

**Fig 10:**
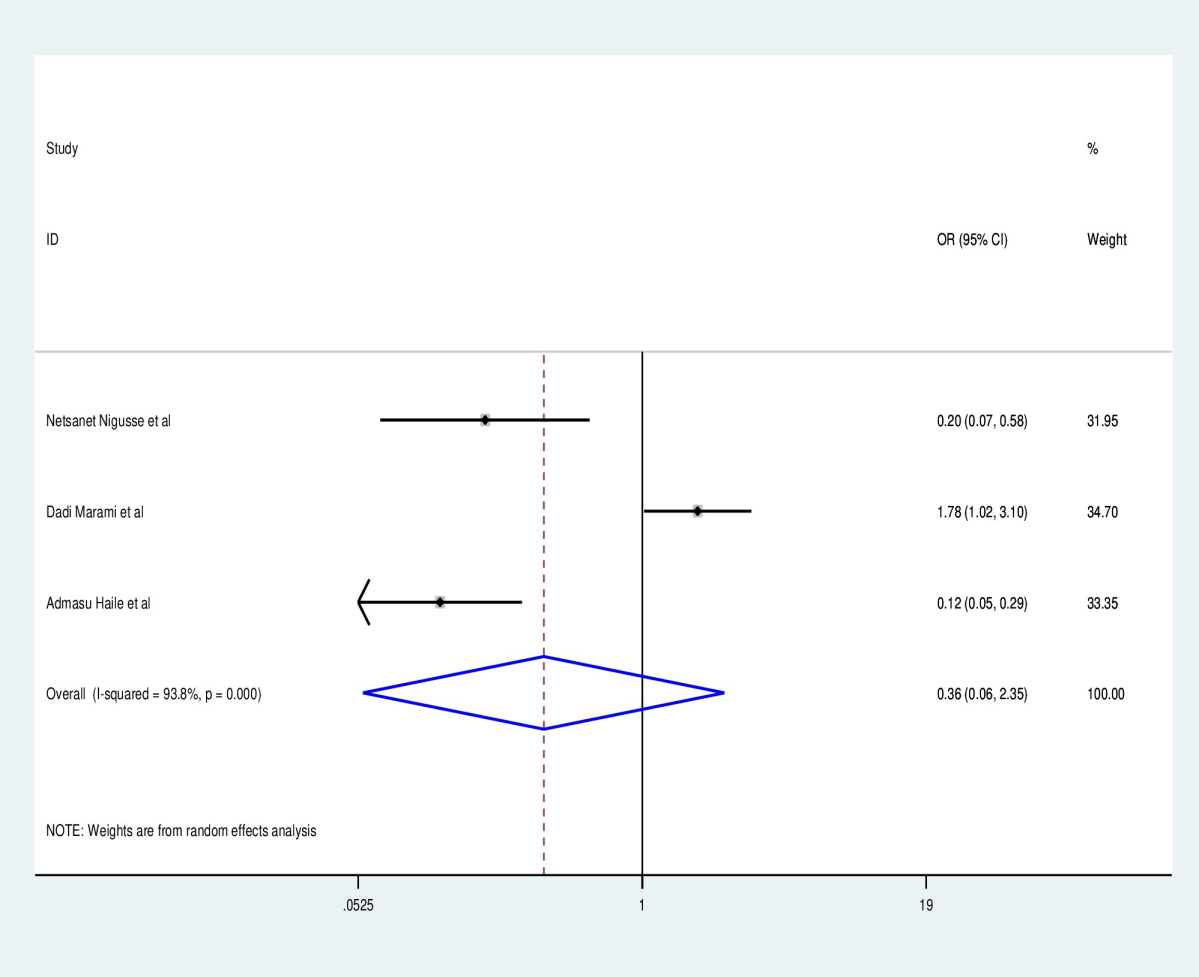
Factor analysis over diabetes mellitus.

Those who have never had a catheterization have a lower risk of obtaining a UTI than those who have (OR: 0.35, 95% CI: 0.06, 1.86) with statistically significant heterogeneity across studies (I^2^: 77.3%, P = 0.01) (Fig 8). Participants in the study who did not have a history of diabetes millitus had a lower risk of UTI than those who did (OR: 0.84, 95% CI:0.12, 0.597), with statistically significant heterogeneity across studies (I^2^:0.84, P = 0.001) (Fig 9). In addition to the foregoing, research participants with CD4 counts larger than 200 had a lower risk of UTI than those with CD4 counts less than 200 (OR:0.36, 95% CI: 0.06, 2.35) with statistically significant heterogeneity (I^2^:93.8%, P = 0.001) (Fig 10).

## Discussions

Urinary tract infection is still a serious public health issue in impoverished nations, including Ethiopia. Its prevalence is fueled by human immunodeficiency virus infection, which is a significant health issue among these populations. About 7 studies with 2257 research participants were included in this systematic review and meta-analysis. These researches took place between 2013 and 2020. All of the studies were observational (cross-sectional), and the study participants were chosen using a simple random, systematic random, and convenience sampling approach. The sample size for each study ranged from 165 to 465 people.

The pooled prevalence of UTI among people living with HIV in Ethiopia was estimated to be 12.8% (95% CI: 10.8–14.79, I^2^ = 50.7%). This is consistent with the 12.3% figure observed in a research in Northern Tanzania [33].

The finding of our study is lower than study conducted in cameroon (67.4%) [34], 27.7% in Nigeria [35] and other study in nigeria 25.3% [36], 40.5% in Nigeria [37]. This could be because in previous studies, nearly half of the study participants [34] had less than 200 CD4 counts, whereas in our study, only one fifth of the study participants had less than 200 CD4 counts, indicating that our study participants’ immune status was relatively intact compared to the previous one [38]. Study participants with excellent immunity are less likely to get a urinary tract infection than those who are immunocompromised [26]. As a result of this, the magnitude of urinary tract infection in our study is less than the previous one.

In addition to the foregoing, this could be because in the previous study, three fifths of the study participants [35] were female, whereas in our study, only about two-fifths of the study participants were female, indicating that the majority of the study participants in our study were males, who have a lower risk of infection than females (or higher in females because of pathogen entry is promoted by sexual activity, anatomy (shorter urethra, and closer proximity of the anus to the vagina), reproductive physiology (pregnancy)) indicating that our study participants were less prone to infection [39–41]. As a result, our study found that the burden of urinary tract infection was lower than the previous one.

Here in our study, those males who are living with HIV have a lower risk of UTI than females (OR:0.35, 95% CI: 0.14, 1.02), although the difference is not statistically significant. It had statistically significant association [26], Participants with CD4 counts more than 200 showed a decreased risk of UTI than those with CD4 counts less than 200 (OR:0.36, 95% CI: 0.06, 2.35) but not statistically significant association in our study although having statistically significant association in a study conducted in Cameroon [34]. In addition to the above, those study participants who have never had a catheterization have a lower risk of obtaining a UTI than those who have (OR: 0.35, 95% CI: 0.06, 1.86) because Catheters put into the bladder cause nosocomial urinary tract infection (UTI) by allowing direct inoculation of microorganisms into the bladder during insertion or during post-insertion manipulation of the catheter or its drainage system. Furthermore, by causing mucosal irritation and providing a biofilm surface for bacterial adhesion, these devices encourage colonization [42], although having statistically significant association. Finally, our study showed that participants who did not have a history of diabetes millitus had a lower risk of UTI [43] than those who did (OR: 0.84, 95% CI:0.12, 0.597), although having statistically significant association.

### Limitation of this study

As a result of the study’s findings, the study participants were drawn from seven studies in Amhara, Oromia, Addis Ababa, and SNNRP, suggesting that they may not accurately reflect the country’s public health problem. Because we included study publications written in English, the number of people affected may be lower.

## Conclusions

In Ethiopia, one out of every eight HIV-positive patients has a possibility of developing UTI. Regardless, we looked for a link between sex, residency, CD4, catheterization history, and DM and UTI, but there was none. To avoid this phenomina, every HIV patient should have a UTI examination in every follow-up.

## Supporting information

S1 File Methodological quality assessment using Newcastle—Ottawa Scale tool. (DOCX)

S2 File The risk of bias assessment tool. (DOCX)

S3 File PRISMA checklist for systematic review and meta-analysis of UTI among people living with HIV in Ethiopia. (DOCX)

S4 File Dataset for catheterization. (DTA)

S5 File Dataset for CD4 count. (DTA)

S6 File Dataset for diabetes mellitus. (DTA)

S7 File Dataset for sex. (DTA)

S8 File Dataset for resident. (DTA)

S9 File (DOCX)

S1 Data (SAV)

## Abbrevations/acronomy

WHO: World Health Organization
UTI: Urinary Tract Infections
ABU: Asymptomatic Bacteriuria
CLSI: Clinical Laboratory Standard Institute
PMTCT: Mother-To-Child Transmission
HMIS: Health Management Information System
HIV: Human Immunodeficiency Virus
IUGR: Intrauterine Growth Restriction
PROM: Premature Rupture Of Membranes
PMCT: Prevention of Mother-to-Child Transmission
ART: Antiretroviral Therapy
JBI: Joanna Briggs Institute
NOS: Newcastle–Ottawa Scale

## References

[1] Ifeanyichukwu I, Emmanuel N, Chika E, Anthonia O, Esther U-I, Ngozi A, et al.: Frequency and antibiogram of uropathogens isolated from urine samples of HIV infected patients on Antiretroviral Therapy. *American Journal of BioScience* 2013, 1(3):50–53.

[2] F B.: Epidemiology of urinary tract infections: incidence, morbidity, and economic costs. *Dis Mon* 2003, 49(2):53–70. doi: 10.1067/mda.2003.7 12601337

[3] Girishbabu RJ, Srikrishna R.and T S.: Asymptomatic bacteriuria in pregnancy. *Int J Biol Med Res*, 2011, 2(3):740–742.

[4] Masinde A, Gumodoka B, Kilonzo A, Mshana S: Prevalence of urinary tract infection among pregnant women at Bugando Medical Centre, Mwanza, Tanzania. *Tanzania journal of health research* 2009, 11(3). doi: 10.4314/thrb.v11i3.47704 20734713

[5] Bigwan E, Wakjissa F: Prevalence of urinary tract infections among HIV patients attending a non-governmental health facility in Jos, Plateau State, Nigeria. *International Journal of Biomedical and Advance Research* 2013, 4(8):528–533.

[6] Zakka S, Oluwatosin OC, Cosmas EU, John S, Rebecca EF, Isa BE: Prevalence of Urinary Tract Infection in HIV Patients on Antiretroviral Drugs in Jos Metropolis, Nigeria. *World* 2018, 3(2):57–60.

[7] Kariuki S, Revathi G, Corkill J, Kiiru J, Mwituria J, Mirza N, et al.: Escherichia coli from community-acquired urinary tract infections resistant to fluoroquinolones and extended-spectrum beta-lactams. *The Journal of Infection in Developing Countries* 2007, 1(03):257–262. 19734602

[8] Wit D, Craven DE., Cabe Mc, R W.: Bacterial infectons in adult patents with the acquired immune defciency syndrome (AIDS) and AIDS related complex. *Am J Med* 1987, 82 (900–906). 10.1016/0002-9343(87)90150-1 3578359

[9] Onorato M, Borucki MJ., Baillargeon G., Paar DP., Freeman DH., et al.: Risk factors for colonizaton or infecton due to methicillin-resistant Staphylococcus aureus in HIV-positve patents: a retrospectve casecontrol study. *Infect Control Hosp Epidemiol* 1999, 20(20–30). 10.1086/501556 9927262

[10] Njoku NHE C. O., Amandi A. N., et al.: Observations on bacterial infection of urinary tract patients. *International Journal of Environmental Health and Human Development*, 1998, 13(2): 785–791.

[11] Moher D, Liberati A, Tetzlaff J, Altman DG: Preferred reporting items for systematic reviews and meta-analyses: the PRISMA statement. *Int J Surg* 2010, 8(5):336–341. 10.1016/j.ijsu.2010.02.007 20171303

[12] Nerurkar A, Solanky P, Naik SS: Bacterial pathogens in urinary tract infection and antibiotic susceptibility pattern. *Journal of Pharmaceutical and Biomedical Sciences* 2012, 21(21).

[13] Ipe DS, Sundac L, Benjamin WH Jr, Moore KH, Ulett GC: Asymptomatic bacteriuria: prevalence rates of causal microorganisms, etiology of infection in different patient populations, and recent advances in molecular detection. *FEMS microbiology letters* 2013, 346(1):1–10. doi: 10.1111/1574-6968.12204 23808987

[14] Peterson J, Welch V, Losos M, Tugwell P: The Newcastle-Ottawa scale (NOS) for assessing the quality of nonrandomised studies in meta-analyses. Ottawa: Ottawa Hospital Research Institute 2011:1–12.

[15] Hoy D, Brooks P, Woolf A, Blyth F, March L, Bain C, et al.: Assessing risk of bias in prevalence studies: modification of an existing tool and evidence of interrater agreement. *Journal of clinical epidemiology* 2012, 65(9):934–939. doi: 10.1016/j.jclinepi.2011.11.014 22742910

[16] Huedo-Medina TB, Sánchez-Meca J, Marin-Martinez F, Botella J: Assessing heterogeneity in meta-analysis: Q statistic or I^2^ index? *Psychological methods* 2006, 11(2):193. doi: 10.1037/1082-989X.11.2.193 16784338

[17] Egger M, Davey-Smith G, Altman D: Systematic reviews in health care: meta-analysis in context: John Wiley & Sons; 2008.

[18] Borenstein M, Hedges LV, Higgins JP, Rothstein HR: A basic introduction to fixed‐effect and random‐effects models for meta‐analysis. *Research synthesis methods* 2010, 1(2):97–111. doi: 10.1002/jrsm.12 26061376

[19] StataCorp L: Stata statistical software (version release 14). College Station, TX: Author 2015, 464:465.

[20] Chaula T, Seni J, Ng’walida N, Kajura A, Mirambo MM, DeVinney R, et al.: Urinary tract infections among HIV-positive pregnant women in Mwanza city, Tanzania, are high and predicted by low CD4+ Count. *International journal of microbiology* 2017, 2017. doi: 10.1155/2017/4042686 28255302 PMC5307130

[21] Awolude OA, Adesina OA, Oladokun A, Mutiu W, Adewole IF: Asymptomatic bacteriuria among HIV positive pregnant women. *Virulence* 2010, 1(3):130–133. doi: 10.4161/viru.1.3.11384 21178431

[22] Widmer TA, Theron G, Grove D: Prevalence and risks of asymptomatic bacteriuria among HIV-positive pregnant women. *Southern African Journal of Epidemiology and Infection* 2010, 25(1):28–32.

[23] Ezechi OC, Gab-Okafor CV, Oladele DA, Kalejaiye OO, Oke BO, Ekama SO, et al.: Prevalence and risk factors of asymptomatic bacteriuria among pregnant Nigerians infected with HIV. *The Journal of Maternal-Fetal & Neonatal Medicine* 2013, 26(4):402–406. doi: 10.3109/14767058.2012.733782 23186370

[24] Ngowi BN, Sunguya B, Herman A, Chacha A, Maro E, Rugarabamu LF, et al.: Prevalence of Multidrug Resistant UTI Among People Living with HIV in Northern Tanzania. *Infection and drug resistance* 2021, 14:1623. doi: 10.2147/IDR.S299776 33911886 PMC8075732

[25] Okechukwu A, Thairu Y: Bacteria urinary tract infection in HIV-infected children and adolescents in Abuja, Nigeria: a cross-sectional study. *African Journal of Clinical and Experimental Microbiology* 2019, 20(4):306–314.

[26] Tessema NN, Ali MM, Zenebe MH: Bacterial associated urinary tract infection, risk factors, and drug susceptibility profile among adult people living with HIV at Haswassa University Comprehensive Specialized Hospital, Hawassa, Southern Esthiopia. *Scientific Reports* 2020, 10(1):1–9. doi: 10.1038/s41598-019-56847-4 32612139 PMC7330024

[27] Debalke S, Cheneke W, Tassew H, Awol M: Urinary tract infection among antiretroviral therapy users and nonusers in Jimma University Specialized Hospital, Jimma, Ethiopia. *International journal of microbiology* 2014, 2014. doi: 10.1155/2014/968716 PMC4009233 24829582

[28] Fenta G, Legese M, Weldearegay G: Bacteriuria and their antibiotic susceptibility patterns among people living with HIV attending Tikur Anbessa Specialized and Zewditu Memorial Hospital ART Clinics, Addis Ababa, Ethiopia. *J Bacteriol Parasitol* 2016, 7(05).

[29] Marami D, Balakrishnan S, Seyoum B: Prevalence, Antimicrobial Susceptibility Pattern of Bacterial Isolates, and Associated Factors of Urinary Tract Infections among HIV‐Positive Patients at Hiwot Fana Specialized University Hospital, Eastern Ethiopia. *Canadian Journal of Infectious Diseases and Medical Microbiology* 2019, 2019. 10.1155/2019/6780354 PMC6381576 30881531

[30] Getu Y, Ali I, Lema T, Belay H, Yeshetela B: Bacteriuria and antimicrobial susceptibility pattern among HIV patients attending ALERT Center, Addis Ababa, Ethiopia. *Am J Health Res* 2017, 5(3):76–82.

[31] Alemu A, Dagnew M, Alem M, Gizachew M: Uropathogenic bacterial isolates and their antimicrobial susceptibility patterns among HIV/AIDS patients attending Gondar University Specialized Hospital, Gondar, Northwest Ethiopia. *J Microbiol Res Rev* 2013, 1(4):42–51.

[32] Hantalo AH, Taassaw KH, Bisetegen FS, Mulate YW: Isolation and Antibiotic Susceptibility Pattern of Bacterial Uropathogens and Associated Factors Among Adult People Living with HIV/AIDS Attending the HIV Center at Wolaita Sodo University Teaching Referral Hospital, South Ethiopia. *HIV/AIDS (Auckland*, *NZ)* 2020, 12:799. 10.2147/HIV.S244619 PMC7708265 33273865

[33] Skrzat-Klapaczyńska A, Matłosz B, Bednarska A, Paciorek M, Firląg-Burkacka E, Horban A, et al.: Factors associated with urinary tract infections among HIV-1 infected patients. *PLOS one* 2018, 13(1):e0190564. doi: 10.1371/journal.pone.0190564 29324763 PMC5764262

[34] Samje M, Yongwa O, Enekegbe AM, Njoya S: Prevalence and antibiotic susceptibility pattern of bacteriuria among HIV-seropositive patients attending the Bamenda Regional Hospital, Cameroon. *African health sciences* 2020, 20(3):1045–1052. doi: 10.4314/ahs.v20i3.7 33402950 PMC7751511

[35] Omoregie R, Eghafona N: Urinary tract infection among asymptomatic HIV patients in Benin City, Nigeria. *British Journal of Biomedical Science* 2009, 66(4):190–193. doi: 10.1080/09674845.2009.11730272 20095127

[36] Rashmi K, RaviKumar K, Bhagyashree H: Asymptomatic bacteriuria in HIV/AIDS patients: occurrence and risk associated with low CD4 counts. *Journal of Evolution of Medical and Dental Sciences* 2013, 2(19):3358–3367.

[37] Kanu A, Mgbajiaka N, Abadom N: Prevalence of urinary tract infection among HIV patients in Aba, Nigeria. *International Journal of Infectious Diseases* 2016, 45:229.

[38] Abraham SN, Miao Y: The nature of immune responses to urinary tract infections. *Nature Reviews Immunology* 2015, 15(10):655–663. doi: 10.1038/nri3887 26388331 PMC4926313

[39] Foxman B: The epidemiology of urinary tract infection. *Nature Reviews Urology* 2010, 7(12):653–660. doi: 10.1038/nrurol.2010.190 21139641

[40] Feitosa DCA, Silva MGd, Parada CMGdL: Accuracy of simple urine tests for diagnosis of urinary tract infections in low-risk pregnant women. *Revista latino-americana de enfermagem* 2009, 17:507–513. doi: 10.1590/s0104-11692009000400012 19820858

[41] Abdullah A, Al Moslih M: Prevalence of asymptomatic bacteriuria in pregnant women in Sharjah, United Arab Emirates. *EMHJ-Eastern Mediterranean Health Journal*, 11 (5–6), 1045–1052, 2005 2005. 16761676

[42] Vergidis P, Patel R: Novel approaches to the diagnosis, prevention, and treatment of medical device-associated infections. *Infectious disease clinics* 2012, 26(1):173–186. doi: 10.1016/j.idc.2011.09.012 22284383 PMC3269005

[43] Nitzan O, Elias M, Chazan B, Saliba W: Urinary tract infections in patients with type 2 diabetes mellitus: review of prevalence, diagnosis, and management. *Diabetes*, *metabolic syndrome and obesity*: *targets and therapy* 2015, 8:129. 10.2147/DMSO.S51792 PMC4346284 25759592

