# Supplementary material for: Magnitude and associated factors of urinary tract infections among adults living with HIV in Ethiopia. Systematic review and meta-analysis
Source: PLoS One. 2022 Apr 1;17(4):e0264732. doi: 10.1371/journal.pone.0264732 (PMC8975107; doi:10.1371/journal.pone.0264732)
Supplement: S1 File — (DOCX) [file pone.0264732.s001.docx]

**Supplementary file 1:** The methodological quality assessment Newcastle - Ottawa Scale tool (NOS)

| **1^st^ author**  **[reference]** | **Criteria** | | | | | | | | |  |
| --- | --- | --- | --- | --- | --- | --- | --- | --- | --- | --- |
|  | **Selection** | | | | | **Comparability** | | **Outcome** | |  |
|  | Study Design | Representativeness of the sample | Sample size | Non –responders | Ascertainment of exposure/risk factor | The study controls for the most important factor | The study control for any additional factor | Assessment of the outcome | Statistical test | Quality score |
| Netsanet Nigusse et *al [20]* | Cross-sectional | A* | A* | B* | B* | - | - | A* | **A*** | **6** |
| Serkadis Debalke,et *al [21]* | Cross-sectional | A* | A* | B* | B* | - | - | A* | **A*** | **6** |
| Genet Molla et *al [22]* | Cross-sectional | B* | B* | B* | B* | - | - | A* | **A*** | **6** |
| Dadi Marami et *al [23]* | Cross-sectional | B* | A* | B* | A* | B* | B* | A* | **A*** | **8** |
| Yemisrach Getu, et *al [24]* | Cross-sectional | A* | A* | A* | B* | - | - | A* | **A*** | **6** |
| Agersew Alemu, et *al [25]* | Cross-sectional | A* | A* | A* | B* | B* | - | A* | **A*** | **8** |
| Admasu Haile et *al [26]* | Cross-sectional | A* | A* | A* | B* | - | - | A* | **A*** | **6** |

Selection: (Maximum 5 stars)
1) Representativeness of the sample: a) Truly representative of the average in the target population. * (all subjects or random sampling) .b) Somewhat representative of the average in the target population. * (nonrandom sampling) .c) Selected group of users.d) No description of the sampling strategy.
2) Sample size:a) Justified and satisfactory. *.b) Not justified.
3) Non-respondents: a) Comparability between respondents and non-respondents characteristics is
established, and the response rate is satisfactory. * .b) The response rate is unsatisfactory, or the comparability between respondents
and non-respondents is unsatisfactory. c) No description of the response rate or the characteristics of the responders and
the non-responders.
4) Ascertainment of the exposure (risk factor): a) validated measurement tool. ** .b) Non-validated measurement tool, but the tool is available or described.* c) No description of the measurement tool.
Comparability: (Maximum 2 stars)
1) The subjects in different outcome groups are comparable, based on the study design or analysis. Confounding factors are controlled. a) The study controls for the most important factor (select one). * b) The study control for any additional factor. *
Outcome: (Maximum 3 stars)
1) Assessment of the outcome: a) Independent blind assessment. **,b) Record linkage. **,c) Self report. *,d) No description.
2) Statistical test:a) The statistical test used to analyze the data is clearly described and appropriate, and the measurement of the association is presented, including confidence intervals and the probability level (p value). *,b) The statistical test is not appropriate, not described or incomplete
