# Supplementary material for: Magnitude and associated factors of urinary tract infections among adults living with HIV in Ethiopia. Systematic review and meta-analysis
Source: PLoS One. 2022 Apr 1;17(4):e0264732. doi: 10.1371/journal.pone.0264732 (PMC8975107; doi:10.1371/journal.pone.0264732)
Supplement: S2 File — (DOCX) [file pone.0264732.s002.docx]

**Supplementary file 2:** The risk of bias assessment tool.

| Corresponding author  [reference] | representation | Sampling | random selection | non-response bias | data collected | case definition | reliability &validity of tool | mode of data collection | length prevalence period | numerator & denominator | the overall risk of bias |
| --- | --- | --- | --- | --- | --- | --- | --- | --- | --- | --- | --- |
| Netsanet Nigusse et *al [20]* | No | No | No | No | Yes | Yes | Yes | Yes | Yes | Yes | Moderate risk |
| Serkadis Debalke,et *al [21]* | Yes | Yes | Yes | Yes | Yes | Yes | Yes | Yes | Yes | Yes | Low risk |
| Genet Molla et *al [22]* | No | No | No | No | Yes | Yes | Yes | Yes | Yes | Yes | Moderate risk |
| Dadi Marami et *al [23]* | Yes | No | No | No | Yes | Yes | Yes | Yes | Yes | Yes | Moderate risk |
| Yemisrach Getu, et *al [24]* | Yes | Yes | Yes | No | Yes | Yes | Yes | Yes | Yes | Yes | Low risk |
| Agersew Alemu, et *al [25]* | Yes | Yes | Yes | Yes | Yes | Yes | Yes | Yes | Yes | Yes | low risk |
| Admasu Haile et *al [26]* | No | No | No | No | Yes | Yes | Yes | Yes | Yes | Yes | Moderate risk |

- **Note**

Risk of bias assessment tool: Yes (low risk); No (high risk)

1. Representation: Was the study population a close representation of the national population?

2. Sampling: Was the sampling frame a true or close representation of the target population?

3. Random selection: Was some form of random selection used to select the sample OR was a census undertaken?

4. Non-response bias: Was the likelihood of non-response bias minimal?

5. Data collection: Were data collected directly from the subjects?

6. Case definition: Was an acceptable case definition used in the study?

7. Reliability and validity of study tool: Was the study instrument that measured the parameter of interest show to have reliability and validity?

8. Data collection: Was the same mode of data collection used for all subjects?

9. Prevalence period: Was the length of the prevalence period for the parameter of interest appropriate?

10. Numerators and denominators: Were the numerator(s) and denominator(s) for the parameter of interest appropriate?

The overall risk of bias scored based on the number of high risk of bias per study: low risk (≥8), moderate risk (5–7), and high risk (≤4)
